# Fiction, Falsehoods, and Few Facts: Cross-Sectional Study on the Content-Related Quality of Atopic Eczema-Related Videos on YouTube

**DOI:** 10.2196/15599

**Published:** 2020-04-24

**Authors:** Simon M Mueller, Valentina N S Hongler, Pierre Jungo, Lucian Cajacob, Simon Schwegler, Esther H Steveling, Zita-Rose Manjaly Thomas, Oliver Fuchs, Alexander Navarini, Kathrin Scherer, Oliver Brandt

**Affiliations:** 1 Department of Dermatology University Hospital Basel Basel Switzerland; 2 Allergy Unit, Department of Dermatology University Hospital Basel Basel Switzerland; 3 Allergy Unit, Division of Respiratory Medicine Department of Paediatrics, Inselspital University of Bern Bern Switzerland

**Keywords:** YouTube, social media, videos, atopic eczema, atopic dermatitis, quality assessment, Global Quality Scale, DISCERN

## Abstract

**Background:**

In recent years, YouTube has become a recognized source of medical information for health care consumers. Although YouTube has advantages in this context, there are potential dangers as videos may contain nonscientific, misleading, or even harmful information.

**Objective:**

As little is known about YouTube as a source of information on atopic dermatitis (AD), we investigated the content-related quality of AD videos and their perception among YouTube users.

**Methods:**

The quality of the 100 most viewed AD videos was assessed by using the Global Quality Scale (GQS) and the DISCERN instrument. Videos were classified as “useful,” “misleading,” and “potentially harmful,” and the correlations of viewers’ ratings (likes) with the GQS and DISCERN scores were assessed.

**Results:**

Among the 100 videos, 68.0% (68/100) and 62.0% (62/100) were of poor and very poor scientific quality, respectively. Additionally, 32.0% (32/100) of the videos were classified as useful, 48.0% (48/100) were classified as misleading, and 34.0% (34/100) were classified as potentially harmful. Viewers’ ratings did not correlate with the GQS and DISCERN scores. Overall, 50.0% (50/100) of the videos were posted by private individuals and promoters of complementary/alternative treatments, 42.0% (42/100) by therapeutical advertisers, and only 8.0% (8/100) by nonprofit organizations/universities.

**Conclusions:**

Our study demonstrated that two-thirds of the videos analyzed were below acceptable medical quality standards and that many videos were disseminating misleading or even dangerous content. Subjective and anecdotal content was overrepresented, and viewers did not appear to be able to distinguish between high- and low-quality videos. Health promotion strategies by professional medical organizations are needed to improve their presence and visibility on YouTube.

## Introduction

Atopic dermatitis (AD), also known as atopic eczema, is the most common chronic inflammatory skin disease during childhood [1] and is characterized by recurrent itchy eczematous lesions [2]. Globally, up to 20% of children and 3% of adults are affected by this condition [2]. Patients experience not only the disease itself (eg, sleep deprivation due to night-time itching), but also the stigma associated with its visibility to others. As a result, patients with AD are often frustrated and embarrassed, which may lead to stress and perpetuation of an itch-scratch cycle, eventually worsening the condition [3]. Consequently, patients and their families frequently report a low health-related quality of life [3,4]. Considering that a high proportion of patients with skin diseases show high interest in online searches [5], patients with AD may be particularly tempted to seek information about their condition from the internet or social media for the aforementioned reasons. YouTube is such a video-based social media platform that allows users to communicate and share their disease burden and individual experience through videos, which can receive comments [6]. It is currently the second most accessed website worldwide [7], attracting approximately one-third of all internet users [8]. YouTube has an increasing number of videos containing medical information [9-11], which may disseminate inaccurate details owing to the lack of quality control and may cause severe health consequences [9,12]. Although several studies have demonstrated that YouTube is highly accessed as a source of information on dermatological topics [13-22], little is known about YouTube as a source of information on AD. This may be surprising given the high prevalence of this condition and the assumption that the populations most affected (children, adolescents, and their parents) typically belong to the age group of “digital natives” (persons born from 1980 onward) [23]. A previous publication indicated that YouTube videos are indeed highly accessed, commented, and shared in connection with AD and that many of the videos posted provide misleading guidance [24]. However, to the best of our knowledge, there have been no in-depth analyses of the topics posted, quality of medical content, upload sources, and ratings by viewers. Therefore, the objectives of this study were as follows: (1) identify the upload sources, common topics, and YouTube categories of the 100 most viewed videos; (2) investigate the content-related quality of YouTube videos as a source of information on AD by applying two different score instruments; (3) correlate viewers’ ratings with our quality assessment findings; and (4) point out strategies for interventions that increase the quality of AD videos and medical content generally uploaded to YouTube and other social media platforms.

## Methods

### Data Collection

In this cross-sectional study, YouTube was searched on April 18, 2018, using the term “atopic eczema” with the following filter settings: “English UK” (language), “United Kingdom” (country), and “Video” (type). Thereafter, videos were sorted by view count, and the duration, upload date, title, URL, view count, uploader identity, likes/dislikes, category, license type, and origin country were recorded and analyzed. In August 2018, videos were reviewed in more detail. Although categories were re-evaluated and refined, we did not update or change numbers and video rankings. We excluded videos having poor technical quality, duplicate videos, and nonEnglish videos, unless English subtitles were displayed. Similar to many previous studies about YouTube, we limited our analysis to the 100 most viewed videos (Multimedia Appendix 1), as it has been demonstrated that videos rated lower than this have insignificant view counts with a minor impact on the analysis [22,25-28].

### Creation of Content Topics

After gathering data, videos were categorized into themes according to the actual content presented irrespective of the titles. The following 12 categories were created: “education,” “topical treatment,” “systemic treatment,” “complementary and alternative medicine (CAM),” “food, nutrition, and diet,” “bathing/wet wrapping,” “ultraviolet (UV) treatment,” “irritants (clothing, sweat, heat, and allergens),” “stress prevention,” “therapeutical advertisement,” “unclear topic,” and “other topics.” Therapeutical advertising included both nonpharmaceutical and pharmaceutical advertising.

### Quality Assessment

**Figure 1:**
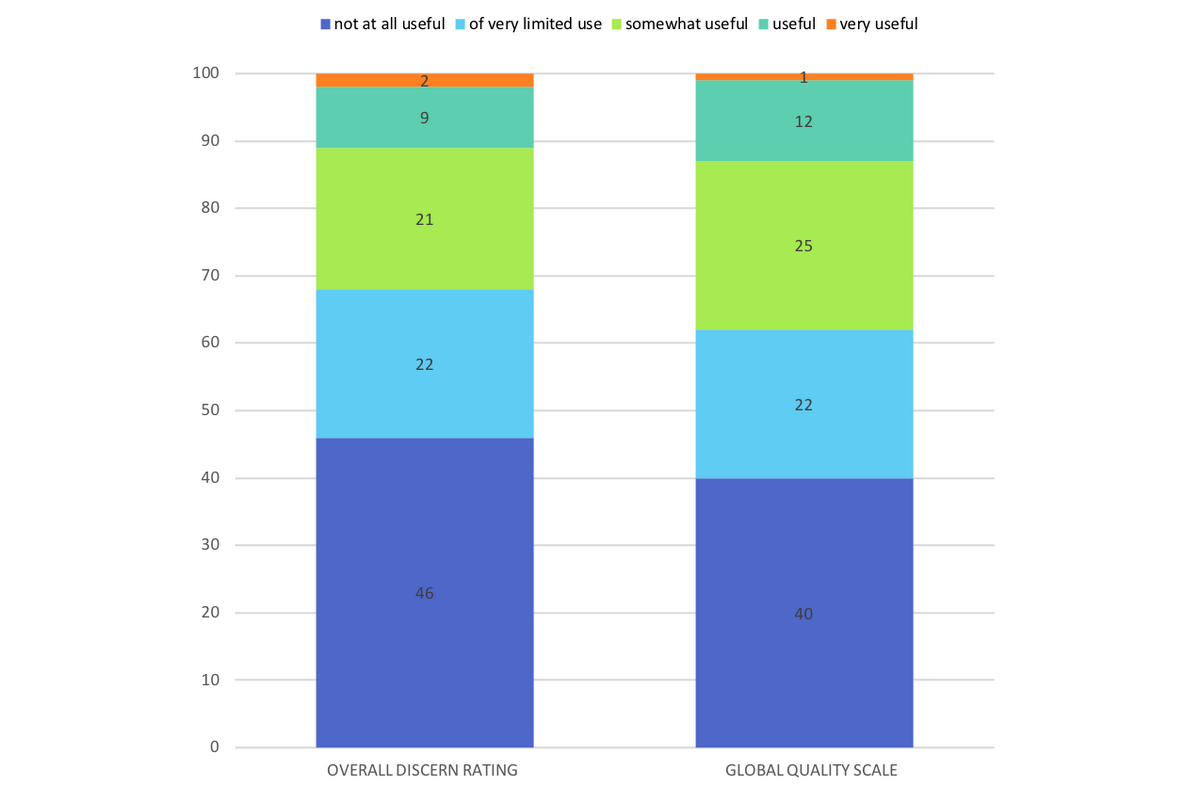
Comparison of the overall quality and usefulness of the identified videos (n=100) for patients seeking health-related advice, using the DISCERN instrument and the Global Quality Scale. The overall rating of DISCERN refers to question 16 of this tool, which is based on a 5-point scale (Multimedia Appendix 2).

The two frequently used quality assessments DISCERN (name of the instrument) and Global Quality Scale (GQS) were applied to evaluate the medical quality of the posted videos [25,29-33]. DISCERN measures a video’s quality of information about treatment choices. It includes 16 questions addressing the reliability (questions 1-8), quality of health information (questions 9-15), and overall quality (question 16) of the videos. To each of these questions 1-5 points were assigned [31,34,35] (Multimedia Appendix 2). The GQS is based on a 5-point scale measuring the content-related quality of a video, its flow, and its value as a source of information for medical laypeople [14,25,29,30,36,37] (Multimedia Appendix 3). The classification shown in Figure 1 (1=not at all useful, 2=of very limited use, 3=somewhat useful, 4=useful, and 5=very useful) was adopted from the report by Qi et al [14]. As both DISCERN and the GQS are based on a 5-point scale, the same classification was used. In addition, videos were classified as “useful” or “misleading,” where useful videos had scientifically correct and accurate information about any aspect of the disease and misleading videos had scientifically unproven or inaccurate information according to currently available scientific evidence (eg, unfounded claims about pathogenesis and treatment with unproven dietary, herbal, or alternative therapy or negative portrayal of evidence-based treatment) [30,32]. If a video could not be assigned to one of these groups, it was automatically classified as “neither nor.” Further, misleading videos were subdivided into “potentially harmful” and “not harmful.” To assess the role of background music in terms of video popularity, we performed correlation calculations with the numbers of likes and views. Regarding all features, in case of different assessments by the analyzing dermatologists, the corresponding videos were re-evaluated and arbitrated by the principal investigator.

### Statistical Analysis

Analyses involving descriptive statistics and Spearman rank correlation coefficients for the numbers of likes/dislikes and the DISCERN and GQS scores were performed using IBM SPSS statistics, version 22.0 (IBM Corp, Armonk, New York). For evaluation of the degree of agreement among reviewers (seven experienced dermatologists listed as authors, except for ZRM, OF, and AN) regarding the videos, Cohen κ coefficients and intraclass correlation coefficients were calculated.

## Results

### View Count, Duration, Upload Sources, Categories, and Topics

The 100 most viewed videos garnered a total of 8,527,624 views and had a total duration of 7 hours 52 minutes (average duration of 4 minutes 44 seconds per video). All videos had a standard YouTube license, which allows the use of the videos only after obtaining permission from the author [38]. Most clips were uploaded from the United States (43.0%, 43/100), followed by unknown countries (14.0%, 14/100) and India (12.0%, 12/100) (Multimedia Appendix 4). The category most often used by the upload source was people & blogs (34.0%, 34/100), followed by education (24.0%, 24/100), how-to & style (22.0%, 22/100), science & technology (11.0%, 11/100), nonprofits & activism (7.0%, 7/100), entertainment (1.0%, 1/100), and news & politics (1.0%, 1/100) (Multimedia Appendix 5A). The most frequent topic (a video can cover more than one topic) was topical treatment (55.0%, 55/100), followed by education (38.0%, 38/100; eg, pathogenesis, risk factors, and instructions for wet-wrapping techniques), food, nutrition, and diet (28.0%, 28/100), CAM (26.0%, 26/100), bathing/wet wrapping (26.0%, 26/100), systemic treatment (15.0%, 15/100), unclear topic (15.0%, 15/100), other topics (13.0%, 13/100), irritants (12.0%, 12/100; clothing, sweat, heat, and allergens), therapeutical advertisement (10.0%, 10/100), UV treatment (5.0%, 5/100), and stress prevention (4.0%, 4/100) (Multimedia Appendix 5B).

Regarding information presenters and upload sources of videos on AD (a video can cover more than one category), the most common were YouTube users with personal experiences and promoters of CAM (50.0%, 50/100), followed by alleged patients (49.0%, 49/100), therapeutical advertisers (42.0%, 42/100), dermatologists or scientists (32.0%, 32/100), health information websites and eczema associations (21.0%, 21/100), nonprofit organizations and universities (8.0%, 8/100), and television/media (6.0%, 6/100) (Multimedia Appendix 6). Videos featuring only patients had approximately 421,181 views, whereas those featuring only dermatologists/scientists had 120,736 views. Background music was used in 51.0% (51/100) of all videos, and these had 823,924 views and 3,481 likes. Background music-containing videos accounted for 53.01% (823,924/1,554,155) of the total view count and had 42.40% (3,481/8,210) of all likes. For none of the videos, YouTube statistics were accessible, whereas the comment function was disabled in only 6.0% (6/100) of the videos.

### Quality Assessment and Correlation With Likes/Dislikes

Overall, 32.0% (32/100) of the videos were classified as useful, 48.0% (48/100) were classified as misleading, and 20.0% (20/100) were classified as neither nor. Among those classified as misleading, 71% (34/48) were considered potentially harmful because of possible mechanical or chemical injury or inadequate dietary recommendations and 29% (14/48) were considered not harmful (Multimedia Appendix 7A). Regarding nonscientific and potentially harmful videos, 37% (15/41) suggested unnecessary diets, 19% (8/41) discredited conventional medicine and physician advice, 15% (6/41) made unrealistic promises, 17% (7/41) did not limit the use of UV treatments, topical steroids, antibiotics, or cold packs, 7% (3/41) promoted the use of unscientific and potentially harmful procedures, and 5% (2/41) recommended topical use of potentially harmful substances (Multimedia Appendix 7B). The mean durations of useful and misleading videos were 6 minutes 2 seconds and 3 minutes 39 seconds, respectively. Regarding the view count, we excluded the most viewed video from further analysis, as it was a pharmaceutical advertisement with disabled like/dislike and comment functions, accounting for approximately 81.78% (6,973,469/8,527,624) of all views. In total, misleading videos had 870,012 views, including 789,073 views for potentially dangerous content, whereas useful videos had only 528,352 views, resulting in a misleading/useful video ratio of 1.65 (870,012/528,352).

**Figure 2:**
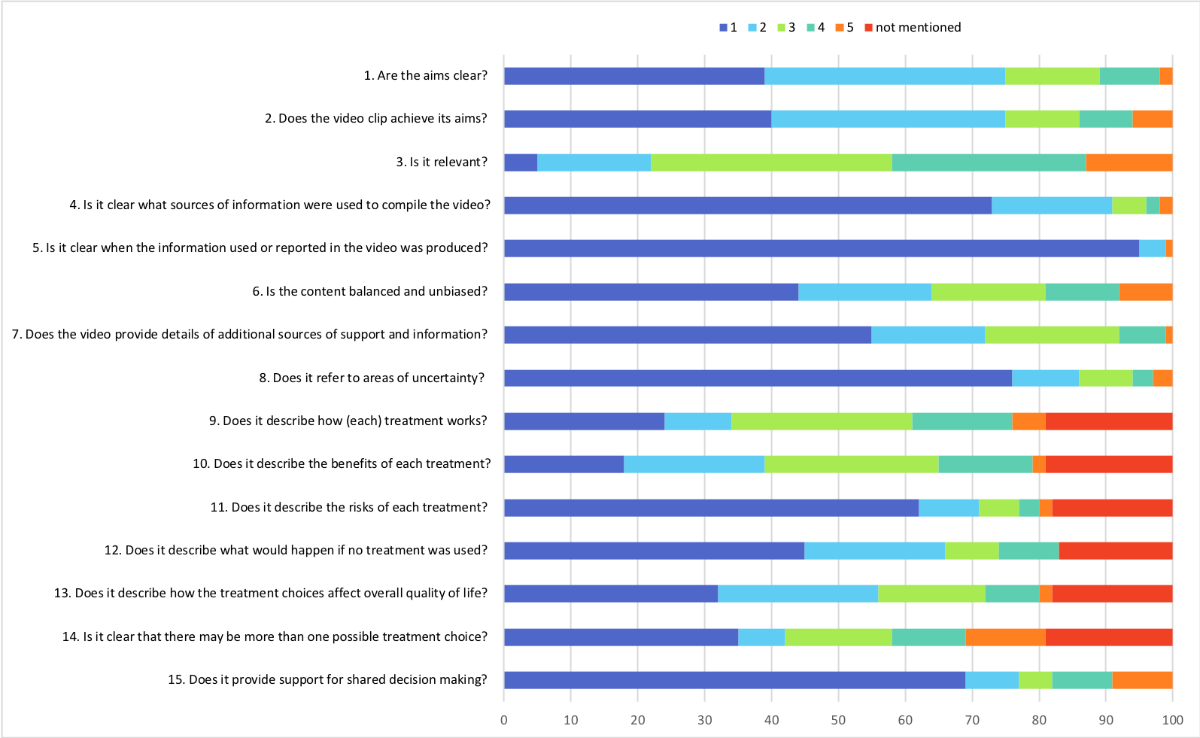
Detailed analysis of the DISCERN questionnaire (questions 1-15) used to rate the identified videos (n=100). The instrument uses a 5-point scale ranging from no to yes, where 1 means “no” (the quality criterion has not been fulfilled at all), 2-4 means “partially” (the quality criterion has been fulfilled to some extent), and 5 means “yes” (the quality criterion has been completely fulfilled).

The ratings achieved with DISCERN and the GQS were consistent, yielding the categorizations shown in Figure 1. With regard to the quality of videos, the mean overall DISCERN and GQS scores were 1.99 (SD 1.09) and 2.12 (SD 1.09), respectively, and therefore, quality was generally low, as the scores range from 1 to 5, with 5 being the highest value. Detailed analysis of the DISCERN questionnaire revealed that the major shortcomings were lack of details about the source of the presented information, unaddressed areas of uncertainty as well as risks of the presented therapy, and failure to recommend shared decision-making (Figure 2). The intraclass correlation coefficients calculated for DISCERN and the GQS were 0.97 and 0.95, respectively, indicating excellent interrater reliability when using these tools.

The total numbers of likes and dislikes for the videos were 8210 and 737, respectively, yielding a like/dislike ratio of 11.14 (8210/737). In three videos, the like/dislike function was disabled. Viewers’ ratings did not correlate with the DISCERN and GQS scores (Spearman correlation ρ=0.12, *P*=.25 and ρ=0.17, *P*=.08, respectively), indicating that viewers were unable to adequately rate the quality of the videos.

## Discussion

### Overview

Patients with AD have a high motivation to conduct online searches, and this usually correlates with disease burden [39,40]. YouTube offers a wide range of dermatology-related videos [13], and little is known so far about the content, upload sources, topics, and scientific quality of these videos. Hitherto, no data exist on whether viewers’ ratings correlate with the quality of the medical information provided.

This study revealed that nearly half of the 100 most viewed AD-related videos on YouTube, with more than 8.5 million views, disseminated misleading information. This finding is consistent with the findings of our previously published study [22] and several other surveys investigating the content-related quality of health-related YouTube videos [12,41,42].

### Comparison With Prior Work

Freemyer et al [24] thoroughly analyzed 128 videos that were gathered by screening the first two result pages of each of the five different search terms (AD, eczema, eczema tips, eczema cure, and eczema treatment) they used for AD. In contrast to our study, they distinguished between “useful,” “useful-personal,” “misleading,” and “misleading-personal” information but not between “harmful” and “neutral” information, and therefore, they found that only 34.4% (44/128) of the videos were misleading. A reason for the markedly lower percentage of misleading videos may be the different search terms used to identify the respective videos. Although a study from 2016 showed that AD is the most commonly used term among scientific publications and studies [43], patients use the nonspecific term eczema to refer to AD [44]. As the purpose of this study was to investigate the video sample patients would come across when seeking information online, we decided to use the term atopic eczema to search YouTube. In contrast to the approach by Freemyer et al [24]*,* we additionally sorted the search results by view count to obtain a video sample of high relevance owing to large viewership. Another aspect that makes direct comparison with our study nearly impossible is the absence of quality assessment tools in the mentioned study.

### Videos of Good or Excellent Quality are Rare

According to our evaluation using DISCERN and the GQS, only 11.0% (11/100) and 13.0% (13/100) of the videos, respectively, were of good or excellent quality with unbiased evidence-based or at least science-based information. Similar results were obtained in our recently published study investigating YouTube videos on psoriasis [22]. Additionally, data by Freemyer et al [24] demonstrated that health care organizations, universities, and dermatologists are clearly underrepresented on YouTube in the context of AD.

### YouTube Users Prefer Low-Quality Videos Over High-Quality Videos

Factual and informative videos are rare, and they lack popularity, as illustrated by the lower number of likes compared to those of poor-quality videos. Our previous study and many other dermatological and nondermatological studies have come to similar conclusions regarding this phenomenon [14,22,41,45]. It remains unclear why the general population tends to prefer low-quality videos over high-quality videos. Biggs et al [46] suggested that the duration of the videos might be a relevant issue. Similar to the findings in their study (mean useful video duration: 14 minutes 47 seconds; mean misleading video duration: 4 minutes 37 seconds), we found that longer videos were associated with higher quality. In our study, useful videos were nearly twice as long as misleading videos (mean useful video duration: 6 minutes 2 seconds; mean misleading video duration: 3 minutes 39 seconds), which could indeed be enough time to put off viewers looking for quick answers. Additional reasons could be that viewers specifically search for alternative content, which is usually present in videos of lower quality, and that academic videos may be less sensationalized and thus less attractive to laypersons [22,37].

### Patients Prefer Advice From Fellow Patients Rather Than Physicians

Interestingly, one or more patients appeared in 49.0% (49/100) of all videos regardless of whether the video was produced by them. Of note, only 32.0% (32/100) of all videos featured a dermatologist or scientist. Analyzing the separate view count, we found that videos featuring only patients had nearly 3.5 times more views as compared with videos featuring only dermatologists/scientists (only patients: 421,181 views; only dermatologists/scientists: 120,736 views). Smith et al [47] showed that 70% of patients with chronic conditions reported experiencing one or more health care–related frustrations, such as the feeling of not being understood or taken seriously (ie, lack of empathy by the physician). Therefore, patients might follow the advice of fellow patients with the same disease rather than the instructions of health care professionals. This decision is obviously frequently made despite poor video quality.

### Potentially Harmful Content

In our study, 34.0% (34/100) of the analyzed videos contained potentially harmful information. For instance, patients with AD were encouraged to not only follow unnecessary diets, such as avoid dairy or gluten, but also use topical treatments (eg, cold packs) and phototherapies without any detailed information about the duration of application or potential risks. Furthermore, conventional medicine and physician advice were discredited in various ways, while promising a fast and easy cure at the same time with the suggested therapies. Such advice was often provided by therapeutical commercials that tried to sell their products, as well as by patients who reported personal negative long-term experiences with Western conventional medicine and eventually found salvation in alternative treatments. Interestingly, these testimonials were frequently uploaded from India (accounting for 12.0% [12/100] of all videos investigated) and typically showed parents being enthusiastic about a traditional practice that healed their children who had atopic eczema.

### Complementary and Alternative Medicine is a Hot Topic for YouTube Users

The confidence and high interest in CAM described above are comprehensible considering that 70%-80% of India’s population is dependent on traditional systems for financing health care [48]. However, this enthusiasm about CAM appears to be shared by the rest of the world as well, because 50.0% (50/100) of the AD-related videos were uploaded by CAM promoters and YouTube users sharing their personal experiences. Reddy et al [37] recently reported the similarly dominant and controversial role of CAM in YouTube videos on food allergy. This may not be surprising considering that the question of food allergies underlying or aggravating AD is important among most affected patients or their parents (particularly in infancy) but is often difficult to answer for clinicians.

### Extensive Advertising

Another astonishing fact is that therapeutical advertisers uploaded 42.0% (42/100) of all videos investigated, although the advertised product did not necessarily appear in the video. As YouTube has become an important marketing platform over the last decade, the advertisement of products with referral links has increased in popularity [49]. Small- and medium-sized companies, in particular, can benefit from social media marketing, as a large number of potential customers can be reached at a relatively low cost [50]. This trend can be an issue for online health information seekers trying to find valid guidance, as commercial advertisements are being disguised as supposedly harmless referral links.

Along with a lack of quality recognition and an intentional search for unconventional content, a lack of entertainment may explain why fewer people are attracted to high-quality videos than to low-quality videos [22]. When examining view count and number of likes with background music as a possible clickbait factor, we found that the majority of likes were generated by videos without background music, suggesting that background music does not markedly increase the popularity of videos and is thus not a suitable strategy to attract people to high-quality videos.

A greater understanding of what kinds of measures attract a high number of viewers is pivotal for enabling health care organizations, universities, and dermatologists to appeal to more patients.

### Potential Interventions to Improve the Presence and Visibility of High-Quality Videos

As the need for content-related high-quality online material is indisputable, dermatology associations, AD self-help organizations, and universities should be encouraged to produce and provide more videos containing evidence-based easy-to-understand information about pathophysiology, clinical manifestations, and therapies for patients with AD and their families, and at the same time, they need to highlight the dangers of non–evidence-based treatment options [22]. Prior to the release of videos, measures for quality assessment (eg, the DISCERN tool) should be applied to ensure high-quality content. In the long term, this approach could result in the neutralization of widely available misleading information on YouTube.

Additionally, professionals need to be aware of the fact that health information seekers mostly choose results that appear on the first page of the search engine [51] when looking for medical content online, and therefore, need to make sure that videos appear there. Investing in consulting services that improve the placement of information in search engines on a website (search engine optimization) could be more efficient than producing additional videos that are placed somewhere beyond the second results page [52]. In addition, it would be advantageous if both social media and search engine providers are encouraged to cooperate with dermatology associations and universities to position medically accurate information near the top of the results page.

Moreover, the use of a Creative Commons license instead of a standard YouTube license could lead to more visits, as has been shown in our previous study [22]. This kind of license allows individuals to use content for their own video clips [53,54]. However, it is advisable to choose a license type that forbids others to change the content in any way or to use it commercially, as misuse that can potentially result in misleading information should be prevented.

Finally, the comment section of misleading videos could be used to insert cross-links to guide viewers to trustworthy videos or websites with evidence-based information. For this approach to work, the comment function must not be disabled by the producer of the video in question, and therefore, unfortunately, this is not a feasible intervention for every video on YouTube.

### Strengths and Limitations

Despite the unmistakable strengths, such as the comprehensive analyses of a high number of videos and the application of two different scoring tools (GQS and DISCERN), this study has some limitations. Although we performed comprehensive analyses, we did not evaluate the comments posted by viewers, which may contain the viewers’ true opinions about a particular video and thus its popularity. Additionally, we did not evaluate the visual design of the videos using a specific tool solely developed for this purpose, making it possible that the impact of the videos on the viewers was not fully captured in this respect. Furthermore, our study is specific for an arbitrary period and therefore only includes the material available at that time. Finally, additional factors, such as instructional design and educational value, contributing to the overall quality of the videos were not assessed in this study. These aspects may certainly be worth investigating in future studies with novel assessment tools. In addition, we would like to mention that besides the assessment tools used in this study, there are other valid methods to measure the information accuracy and content quality of YouTube videos [55].

### Conclusion

This study showed that two-thirds of the videos on AD analyzed were below acceptable medical quality standards and often contained misleading or even harmful information about this common disease. The fact that users tended to rate low-quality videos better than high-quality videos suggests that the majority of users are unable to distinguish between medically credible information and false information. This shows that the numbers of views and likes do not reflect the medical quality of videos. To combat this phenomenon, it is crucial that future studies investigate user motivation for such behavior in order to help medical professionals to develop approaches that contribute to improving medical information on this powerful platform.

## Appendix

Multimedia Appendix 1 Links to the YouTube videos.

Multimedia Appendix 2 DISCERN instrument.

Multimedia Appendix 3 Global Quality Scale (GQS).

Multimedia Appendix 4 Distribution of the videos by country of origin.

Multimedia Appendix 5 (A) YouTube categories, in which the analyzed videos were posted (n=100); (B) Topics presented in the videos (note: a video clip can cover more than one topic).

Multimedia Appendix 6 Distribution of information according to presenters and upload sources (multiple categories may apply to one video). CAM, complementary and alternative medicine.

Multimedia Appendix 7 (A) Classification of the videos (n=100) into “useful,” “misleading,” and “neither nor,” with further subdivision of “misleading” into "potentially harmful” and “not harmful” (not harmful = 48 misleading videos minus potentially harmful videos [14, 29%]) shown as a bar chart (in percentage); (B) Specifications and examples of nonscientific and potentially harmful videos.

## Abbreviations

AD: atopic dermatitis
CAM: complementary and alternative medicine
GQS: Global Quality Scale
UV: ultraviolet light
