# Supplementary material for: Fiction, Falsehoods, and Few Facts: Cross-Sectional Study on the Content-Related Quality of Atopic Eczema-Related Videos on YouTube
Source: J Med Internet Res. 2020 Apr 24;22(4):e15599. doi: 10.2196/15599 (PMC7210495; doi:10.2196/15599)
Supplement: Multimedia Appendix 2 [file jmir_v22i4e15599_app2.pdf]

Multimedia Appendix 2.

DISCERN instrument for evaluating health information of online videos (adapted from Charnock [31]).

Questions	Points
1. Are the aims clear?	1-5
2. Does the video clip achieve its aims?	1-5
3. Is it relevant?	1-5
4. Is it clear what sources of information were used to compile the video?	1-5
5. Is it clear when the information used or reported in the video was produced?	1-5
6. Is the content balanced and unbiased?	1-5
7. Does the video provide details of additional sources of support and information?	1-5
8. Does it refer to areas of uncertainty?	1-5
9. Does it describe how each treatment works?	1-5
10. Does it describe the benefits of each treatment?	1-5
11. Does it describe the risks of each treatment?	1-5
12. Does it describe what would happen if no treatment was used?	1-5
13. Does it describe how the treatment choices affect overall quality of life?	1-5
14. Is it clear that there may be more than one possible treatment choice?	1-5
15. Does it provide support for shared decision making?	1-5
16. Overall rating of the video based on the answers to all of the above questions	1-5
