# Supplementary material for: Fiction, Falsehoods, and Few Facts: Cross-Sectional Study on the Content-Related Quality of Atopic Eczema-Related Videos on YouTube
Source: J Med Internet Res. 2020 Apr 24;22(4):e15599. doi: 10.2196/15599 (PMC7210495; doi:10.2196/15599)
Supplement: Multimedia Appendix 3 [file jmir_v22i4e15599_app3.pdf]

Multimedia Appendix 3.

Global Quality Score (GQS) used for evaluation of video clips (adapted from Singh [30]).

Item characteristics	Points
Poor quality; poor flow of the video; most information missing; not at all useful for patients	1
Generally poor quality and poor flow; some information listed, but many important topics missing; of very limited use for patients	2
Moderate quality; suboptimal flow; some important information adequately discussed, but other information poorly discussed; somewhat useful for patients	3
Good quality and generally good flow; most of the relevant information listed, but some topics not covered; useful for patients	4
Excellent quality and flow; very useful for patients	5
