# Supplementary figures and images for: Fiction, Falsehoods, and Few Facts: Cross-Sectional Study on the Content-Related Quality of Atopic Eczema-Related Videos on YouTube
Source: J Med Internet Res. 2020 Apr 24;22(4):e15599. doi: 10.2196/15599 (PMC7210495; doi:10.2196/15599)

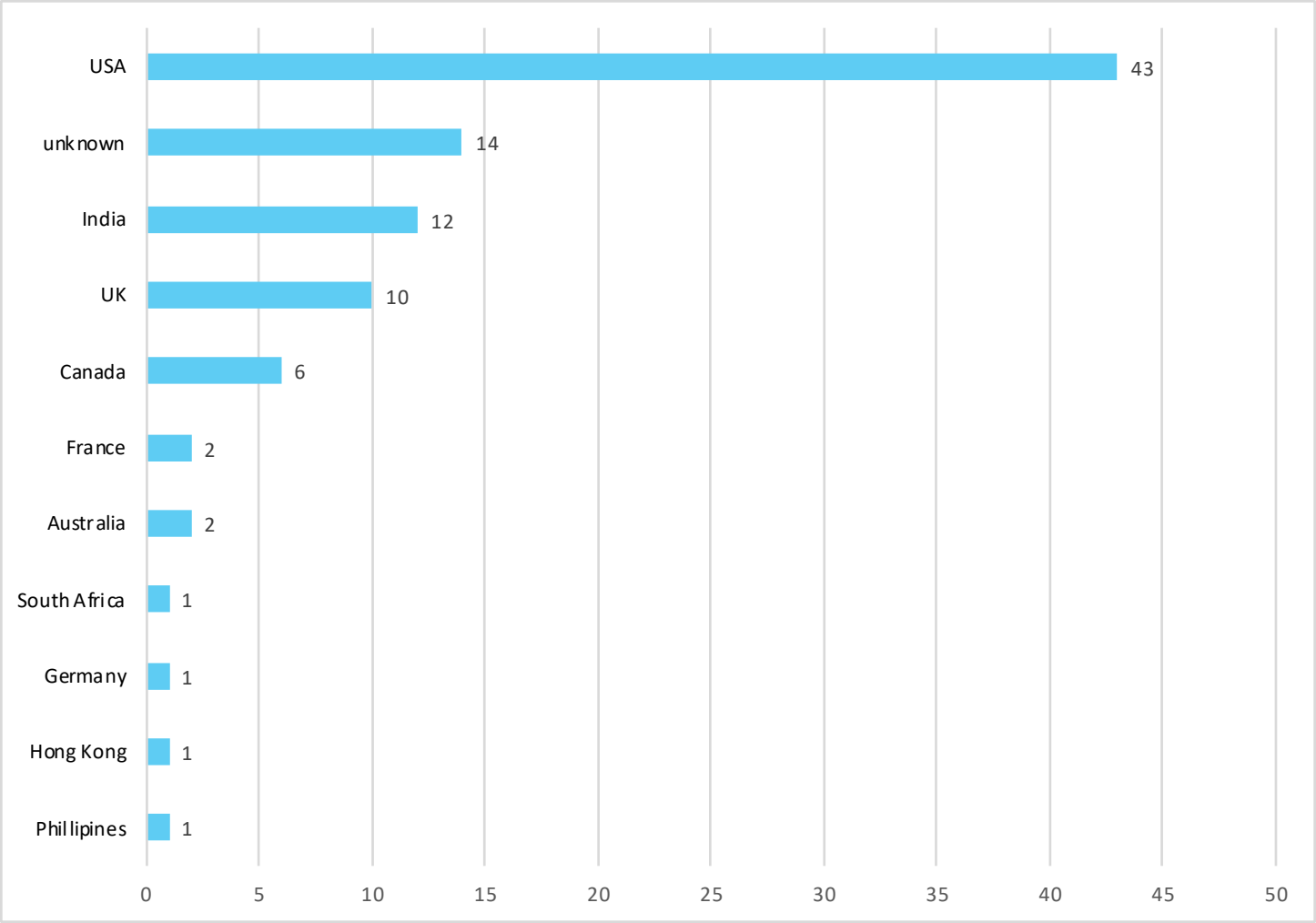

Supplement: Multimedia Appendix 4 [file jmir_v22i4e15599_app4.pdf]

a.)

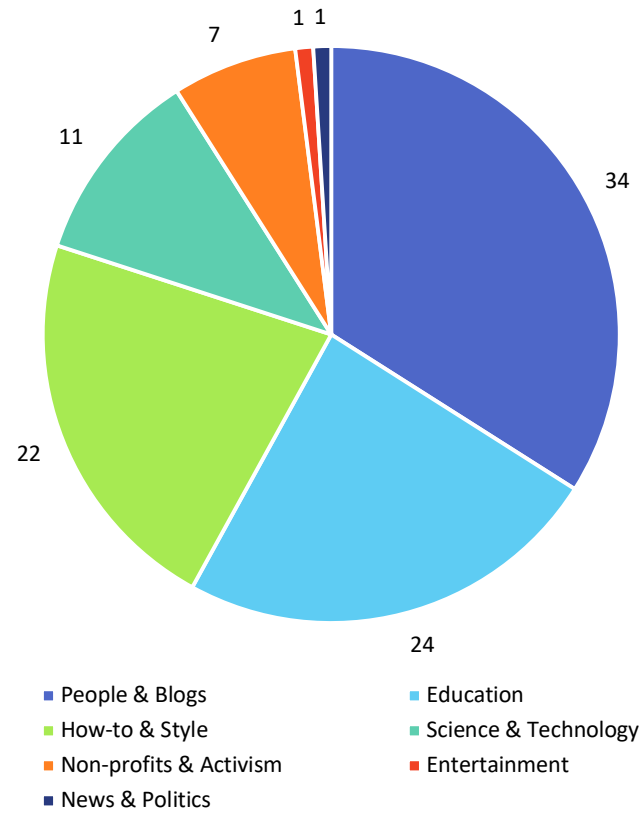

b.)

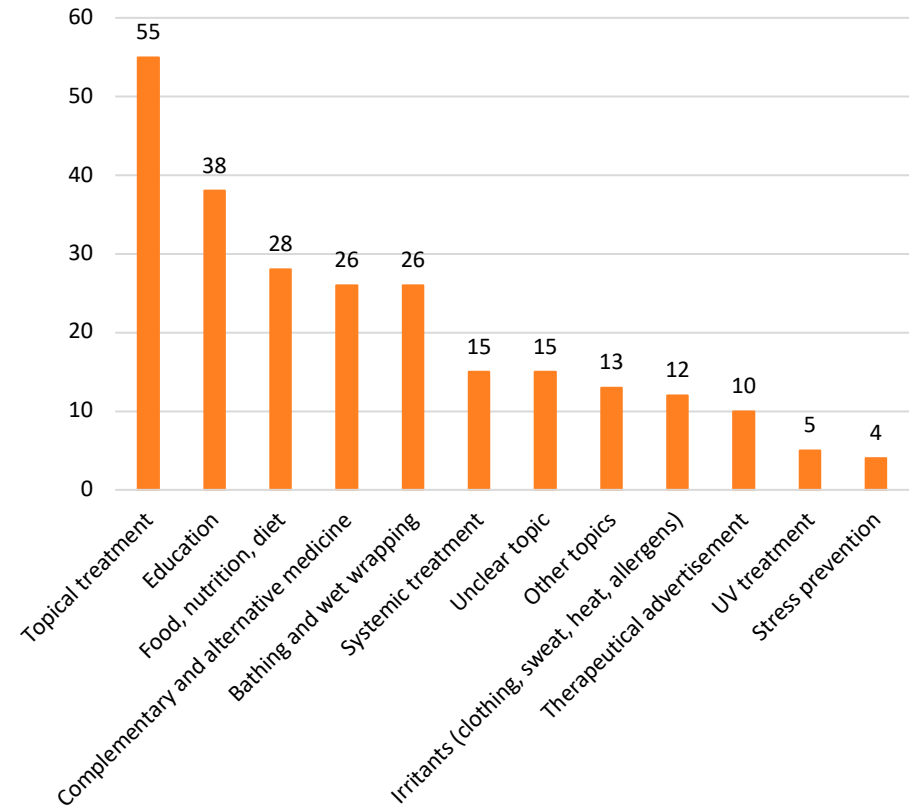

Supplement: Multimedia Appendix 5 [file jmir_v22i4e15599_app5.pdf]

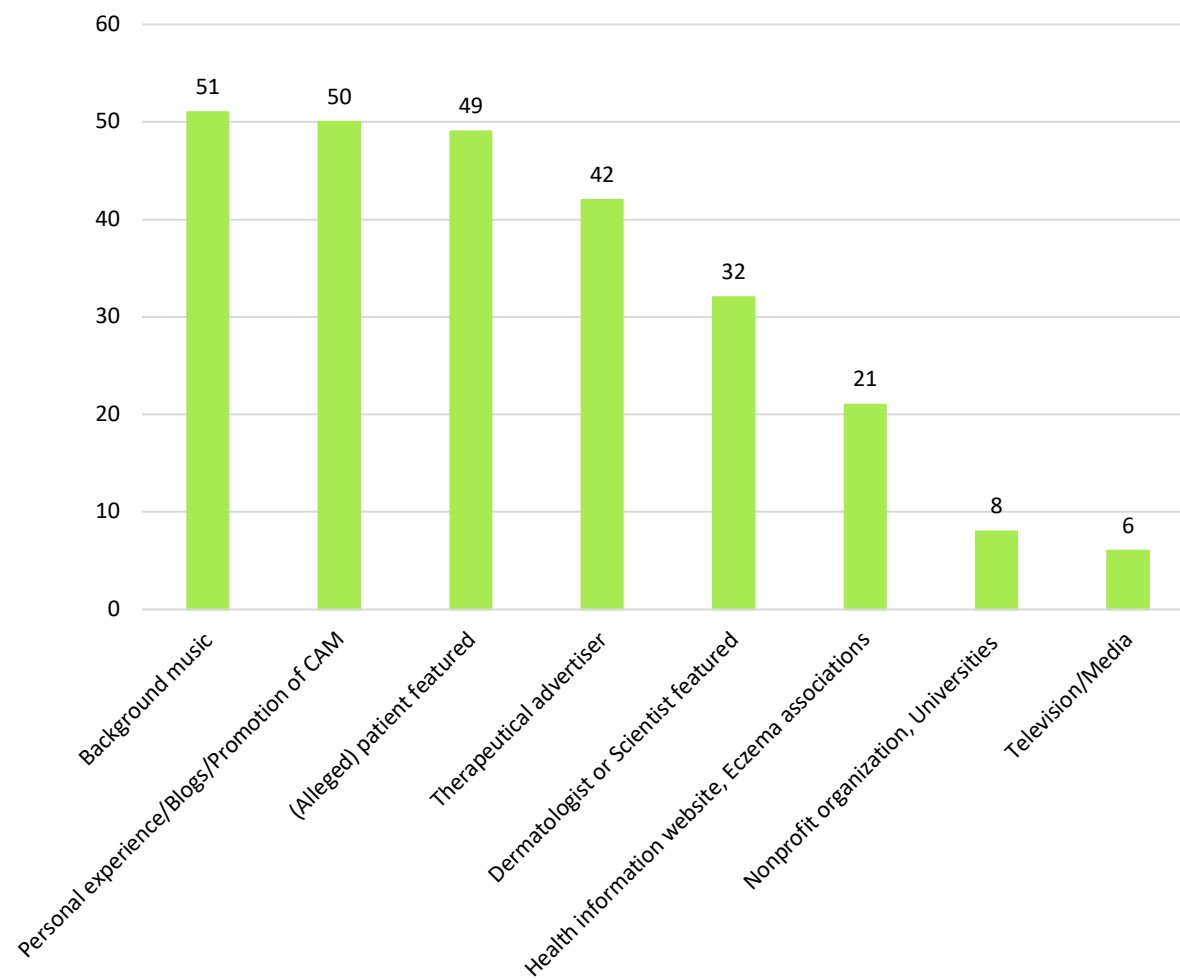

Supplement: Multimedia Appendix 6 [file jmir_v22i4e15599_app6.pdf]

a.)

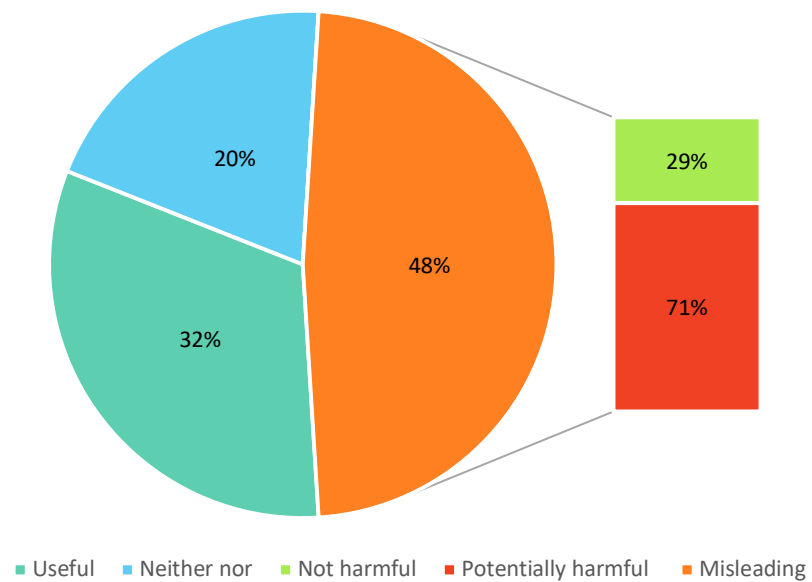

b.)

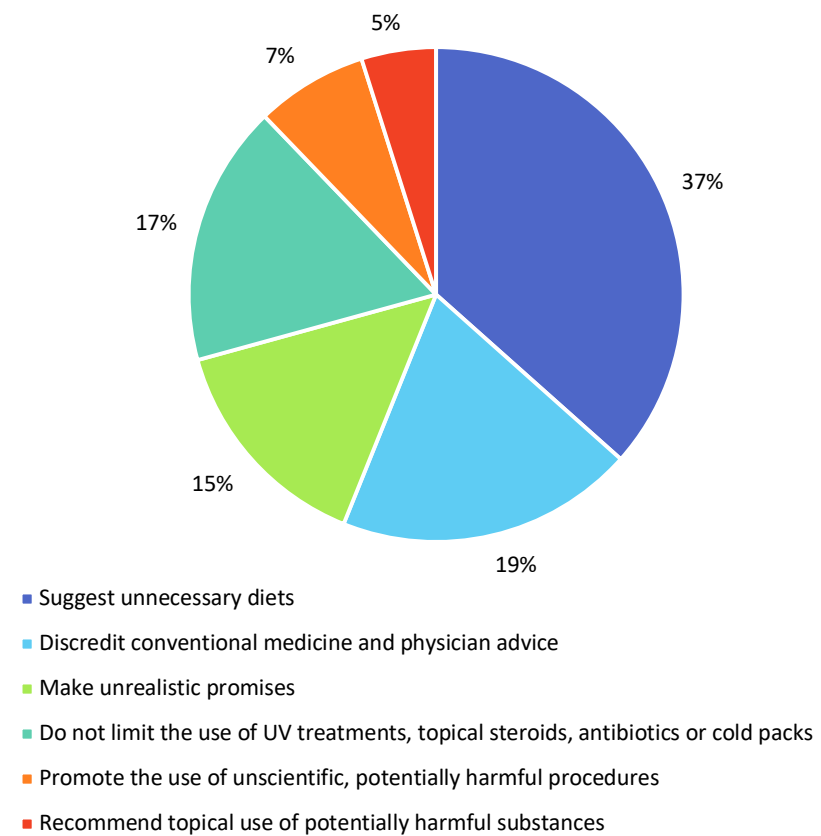

Supplement: Multimedia Appendix 7 [file jmir_v22i4e15599_app7.pdf]
